# Plant invasion and speciation along elevational gradients on the oceanic island La Palma, Canary Islands

**DOI:** 10.1002/ece3.2640

**Published:** 2016-12-27

**Authors:** Manuel J. Steinbauer, Severin D. H. Irl, Juana María González‐Mancebo, Frank T. Breiner, Raquel Hernández‐Hernández, Sebastian Hopfenmüller, Yohannes Kidane, Anke Jentsch, Carl Beierkuhnlein

**Affiliations:** ^1^ Department of BiogeographyBayCEERUniversity of BayreuthBayreuthGermany; ^2^ Section Ecoinformatics & BiodiversityDepartment of BioscienceAarhus UniversityAarhusDenmark; ^3^ Departamento de Botánica, Ecología y Fisiología VegetalUniversidad de La LagunaSan Cristóbal de La LagunaTenerifeSpain; ^4^ Swiss Federal Research Institute WSLBirmensdorfSwitzerland; ^5^ Department of Ecology and EvolutionUniversity of LausanneLausanneSwitzerland; ^6^ Department of Animal Ecology and Tropical BiologyUniversity of WürzburgWürzburgGermany; ^7^ Department of Disturbance EcologyBayCEERUniversity of BayreuthBayreuthGermany

**Keywords:** alien species, altitudinal gradient, colonization, diversification, diversity, endemism, exotic, high‐elevation ecosystems, island biogeography

## Abstract

Ecosystems that provide environmental opportunities but are poor in species and functional richness generally support speciation as well as invasion processes. These processes are expected not to be equally effective along elevational gradients due to specific ecological, spatial, and anthropogenic filters, thus controlling the dispersal and establishment of species. Here, we investigate speciation and invasion processes along elevational gradients. We assess the vascular plant species richness as well as the number and percentage of endemic species and non‐native species systematically along three elevational gradients covering large parts of the climatic range of La Palma, Canary Islands. Species richness was negatively correlated with elevation, while the percentage of Canary endemic species showed a positive relationship. However, the percentage of Canary–Madeira endemics did not show a relationship with elevation. Non‐native species richness (indicating invasion) peaked at 500 m elevation and showed a consistent decline until about 1,200 m elevation. Above that limit, no non‐native species were present in the studied elevational gradients. Ecological, anthropogenic, and spatial filters control richness, diversification, and invasion with elevation. With increase in elevation, richness decreases due to species–area relationships. Ecological limitations of native ruderal species related to anthropogenic pressure are in line with the absence of non‐native species from high elevations indicating directional ecological filtering. Increase in ecological isolation with elevation drives diversification and thus increased percentages of Canary endemics. The best preserved eastern transect, including mature laurel forests, is an exception. The high percentage of Canary–Madeira endemics indicates the cloud forest's environmental uniqueness—and thus ecological isolation—beyond the Macaronesian islands.

## 1 Introduction

Elevational gradients on isolated mountains (especially on oceanic high‐elevation islands) pose a unique opportunity to study important ecological processes, including evolutionary dynamics (Steinbauer et al., 2016) and plant invasion (Alexander et al., 2011; Daehler, 2005). For both processes, oceanic high‐elevation islands offer a unique setting provided by the high rates of species evolved in situ (neo‐endemism; Whittaker et al., 2007).

Islands tend to be more prone to invasion than areas with higher degrees of connectivity on the continent (*island susceptibility hypothesis*, Simberloff, 1995) due to low species richness (i.e., a higher relative ecological opportunity), functional diversity, and reduced competitiveness of species compared to those of the mainland (Sol, 2000). However, recent studies indicate that the island susceptibility hypothesis is not generally valid. Presumably, the isolation of islands (smaller regional species pool) also impedes the establishment of non‐native species (Etherington, 2015; Jeschke, 2008; Vila, Pino, Montero, & Font, 2010), which nowadays mainly occurs via direct anthropogenic vectors.

Introductions of non‐native species are not equally probable along elevational gradients. Accordingly, the majority of non‐native plant species is expected in lower elevations and less extreme habitats, where climatic conditions are more favorable, human disturbances are most frequent, and isolation is lowest (Becker, Dietz, Billeter, Buschmann, & Edwards, 2005; Pauchard et al., 2009). Extreme climatic conditions of high‐elevation ecosystems and low anthropogenic disturbances prevent the establishment of non‐native species (but see exceptions, e.g., Irl, Jentsch, & Walther, 2013; Alexander et al., 2016). Hence, it is probable that the majority of introduced non‐natives are pre‐adapted to mid‐ or low elevations (Pauchard et al., 2009). Accordingly, the low‐elevation alien flora may be the main species pool for the alien flora of high‐elevation ecosystems (McDougall et al., 2011). In contrast to high‐elevation specialists, invasive non‐native species tend to be generalists tolerating a wide range of climatic conditions (Alexander et al., 2011; Higgins & Richardson, 2014), although the distribution pattern in the area of introduction depends on the interaction of traits of the invading species and the environmental conditions (Haider et al., 2010).

On mountainous oceanic islands, low‐elevation ecosystems have larger spatial extent than high‐elevation ecosystems—a typical feature of mountain systems (but see Elsen & Tingley, 2015). Especially tropical and subtropical islands possess a broad climatic range due to windward/leeward effects and thus a comparatively high absolute number of habitats (Hortal, Triantis, Meiri, Thebault, & Sfenthourakis, 2009; Irl et al., 2015). As a consequence, most species on oceanic islands—non‐natives, natives, or endemics—will be found in low‐ and mid‐elevation ecosystems (Zobel et al., 2011). In order to understand invasion processes, however, it is of high interest whether the percentage of non‐native species increases with elevation. The percentage of non‐native species can be seen as an indicator for invasion processes independent of overall richness (c.f., Gillespie, Claridge, & Roderick, 2008) although it is still susceptible to differences in disturbances.

Recent studies suggest that diversification, as indicated by two frequently used indices (i.e., the percentage of single‐island endemic species and the percentage of archipelago endemics species; Emerson & Kolm, 2005; Whittaker et al., 2007), increases with elevation—a pattern which has large‐scale support for islands as well as continental mountains (Steinbauer et al., 2016). However, this trend can be modified on an intra‐insular level by other environmental influences such as different aspects of climate (esp. precipitation; Irl et al., 2015) and should be stronger the more pronounced environmental changes along the elevational gradient are. Additionally, diversification is supported by genetic isolation (and hence a tendency toward speciation) and the availability of ecological opportunities due to low species richness (Hughes & Eastwood, 2006; Steinbauer & Beierkuhnlein, 2010).

Directional ecological filtering along elevational gradients (Alexander et al., 2011) causes isolation of high‐elevation ecosystems (Gillespie, 2004). Particularly on oceanic islands, high‐elevation ecosystems tend to be more isolated from the nearest ecosystems with comparable environment than their low‐elevation counterparts (Steinbauer et al., 2016). The latter are less distant from similar habitats on other islands and on the mainland (Steinbauer, Irl, & Beierkuhnlein, 2013). In consequence, high‐elevation systems form islands within islands (Fernández‐Palacios, Otto, Thebaud, & Price, 2014). Elevational gradients thus provide an excellent natural setting to investigate the effect of isolation on speciation and invasion processes.

Here, we test with a specifically designed field study the following predictions using vegetation surveys along three elevation gradients ranging more than 2000 m on La Palma (Canary Islands). Based on current knowledge, we expect (1) that the vascular plant species richness as well as the number of endemics (endemic to La Palma, endemic to the Canary Islands or shared between Madeira and the Canary Islands, respectively) decreases, while the percentage of endemic species increases with elevation. (2) The absolute number of non‐native species decreases, while the percentage of non‐native species increases with elevation, if the availability of unoccupied habitats at high‐elevation ecosystems significantly enhances the establishment of non‐native species. The contrary is to be expected, if the extreme environmental conditions at high elevations pose a more effective filter for non‐native species than the already bypassed geographical isolation.

## 2 Materials and Methods

### 2.1 Study area

**Figure 1:**
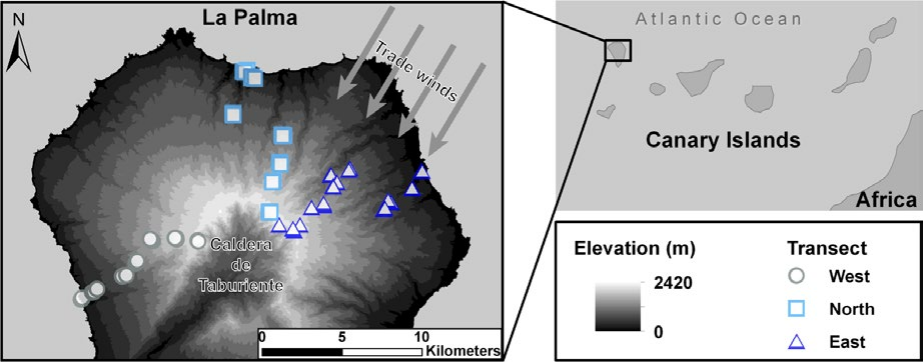
Map of La Palma. La Palma is the northwestern most island of the Canary Islands situated off the coast of Morocco (Africa) in the Atlantic Ocean. The map illustrates the location of the western (circles, *n *=* *21), northern (squares, *n *=* *21), and eastern transects (triangle, *n *=* *24) covering a large elevational gradient of more than 2400 m in detail. Due to the predominant trade wind direction from the northeast, precipitation increases from western to eastern transect, especially at mid‐elevations

La Palma is one of the most western and youngest islands of the Canary archipelago (~1.7 Ma; Carracedo et al., 2002) expressing continuous volcanic activity (Prieto et al., 2009). The island covers 706 km² and reaches a maximum elevation of 2426 m a.s.l. at the Roque de los Muchachos (Figure 1). The northern part of La Palma is comprised by the Caldera de Taburiente complex and is the oldest and steepest part of the island, thus offering also steep environmental gradients (Irl et al., 2015).

Owing to its northwestern position, La Palma receives fair amounts of precipitation in winter, yet extensive dry periods are common on the western side of the island and at high elevations (Irl & Beierkuhnlein, 2011). Besides elevation, the dominating climatic influence factors are humid trade winds regularly blowing from the northeast (Garzón‐Machado, Otto & del Arco Aguilar, 2014) and dividing the island into a humid windward side and a drought and fire‐prone leeward side. Thus, the main precipitation gradient of the island runs along a northeast–southwest axis reaching from the western coast (approx. 120 mm/a) to the high‐elevation northeastern slopes of the Caldera de Taburiente (approx. 1,300 mm/a; AEMET, 2012). Above the cloudbank, a thermal inversion layer exists that impedes an orographic rise of moist air masses eliminating the trade wind influence. Above the thermal inversion layer, high solar radiation and high daily temperature amplitudes are common. There, winter snow appears regularly and even ice storms can occur (Garzón‐Machado et al., 2014).

### 2.2 Sampling design

Three transects for floristic inventory were installed across the complete elevational gradient. One reached the caldera rim from the dry west coast, one from the more humid north coast, and one from the northeast through the laurel forest belt, thus, systematically designed to cover all vegetation zones and the complete precipitation gradient offered by La Palma (Figure 1). Within each transect, we installed plots (10 × 10 m) at preselected elevational levels. Criteria for plot selection were accessibility and a slope not higher that 35°, but always uninfluenced by roads. Along the western and northern transects, we established 21 plots distributed evenly between seven elevation levels spanning 100 m and separated by 300‐m intervals (note: the interval between the second highest and the highest elevation level only measured 200 m, because a maximum elevation of 2,400 m a.s.l. could not be exceeded). Three replicates per level were established, which is in accordance with guidelines for optimal sampling along environmental gradients (Schweiger, Irl, Steinbauer, Dengler, & Beierkuhnlein, 2016). Data sampling on the western and northern transects took place in February 2009. Along the eastern transect, 24 plots distributed across 12 elevation levels with 200‐m intervals were installed. Thus, two replicates were established per level. Data sampling for the eastern transect took place from February to April 2013. All raw data are provided in online Appendix.

We recorded all flowering plant species and classified them as single‐island endemics, archipelago endemics (i.e., endemic to the Canary Islands), and non‐native and nonendemic (including definitely native, possibly native and probably native) according to Acebes Ginovés et al. (2010). In our study, the group of non‐natives consists of all sub‐classes given by Acebes Ginovés et al. (2010), that is, differentiated into introduced species, probably introduced species and introduced invasives.

As the Canary Islands share a high number of endemic species with Madeira, we also classified *Canary–Madeira endemics*. This group includes all the Canary endemics plus the species with a globally restricted distribution range to the Canary Islands and Madeira. The Canary Islands and Madeira currently are located at a distance less than 400 km and were even closer during time periods with lower sea level (Fernández‐Palacios et al., 2011). We further identified a subset of Madeira–Canary endemics by excluding those species that only occurred on the Canaries—thus focusing only on endemic species inhabiting both archipelagos (further on referred to as *Madeira–Canary twins*).

### 2.3 Statistical analysis

The relation between indices (for richness, endemism, and invasion) with elevation was assessed using generalized linear mixed models (R package lme4 version 1.1‐7, Bates, Maechler, Bolker, & Walker, 2015). Full models included *elevation*,* elevation* ^*2*^, *transect* as well as the interactions between transect and elevation as explanatory variables (*y* ~ elevation + elevation^2^ + transect + transect:elevation). In a further step, elevation, elevation^2^, and/or the interaction between transect and elevation was removed if not significantly contributing to the explanatory power of the model. The later was tested using a *F* test (ANOVA) between a model including and one excluding the focal variable (backward selection; Table 1 provides an overview on final model specifications). As vegetation plots are replicated per elevational level, we included a block factor in the random structure of the models to account for this spatial clustering.

**Table 1:** Model structure for different dependent variables

Variable	Model structure	Family	$R_{\mathrm{Marg}}^{2}$	$R_{\mathrm{Cond}}^{2}$
Overall richness	*y* ~ elevation*** + transect^n.sig^ + elevation:transect* + (1|block)	Poisson	.74	.79
Native richness	*y* ~ elevation*** + transect^n.sig^ + elevation:transect* + (1|block)	Poisson	.72	.77
% Natives	*y* ~ elevation* + elevation^2^ * + transect^n.sig^ + (1|block)	Binomial	.81	.81
Archipelago endemics	*y* ~ elevation*** + elevation^2^ ** + transect* + (1|block)	Poisson	.40	.48
% Archipelago endemics	*y* ~ elevation*** + elevation^2^ *** + transect* + (1|block)	Binomial	.71^a^	–
Island endemics	*y* ~ elevation * + transect*** + (1|block)	Poisson	.44	.56
% Island endemics	*y* ~ elevation^2^ * + transect*** + (1|block)	Binomial	.32^a^	–
Canary–Madeira endemics	*y* ~ elevation*** + elevation^2^ * + transect^n.sig^ + (1|block)	Poisson	.59	.59
% Canary–Madeira endemics	*y *~ elevation*** + elev.^2^ *** + transect** + elev.:transect* + (1|block)	Binomial	.66^a^	–
Madeira–Canary twins	*y* ~ elevation*** + elev.^2^ *** + transect^n.sig^ + elev.:transect** + (1|block)	Poisson	.83^a^	–
% Madeira–Canary twins	*y* ~ elevation** + elevation^2^ ** + transect*** + (1|block)	Binomial	.82	.83
Non‐natives	*y* ~ elevation* + elevation^2^ ** + transect** + (1|block)	Poisson	.77^a^	–
% Non‐natives	*y* ~ elevation*** + elevation^2^ * + transect^n.sig^ + (1|block)	Binomial	.50^a^	–

For models marked with an “a” at the $R_{\mathrm{Marg}}^{2}$, pseudo‐*R* ^2^ was calculated after removing the random structure (block effect) from the model as the function *piecewiseSEM* failed to provide pseudo‐*R* ^2^ values for the mixed effect model.

Significance of explanatory variables (based on *F*‐statistics) is indicated with stars (*p *<* *.05*; *p *<* *.01**; *p *<* *.001***). Marginal ($R_{\mathrm{Marg}}^{2}$) and conditional ($R_{\mathrm{Cond}}^{2}$) pseudo‐*R* ^2^ were calculated based on R package *piecewiseSEM* (Lefcheck, 2015).

Poisson error distribution (log‐link function) was used for all richness‐based indices, while binomial error distribution (logit‐link function) was used for percentage values. The binomial error distribution for percentage values has the advantage to account for the fact that a particular percentage value is more precise if it is based on a larger number of observations (here the number of species). Note that percentage of endemic species is calculated excluding non‐native species to get a cleaner signal for diversification. Patterns remain, however, if non‐natives were included.

Goodness‐of‐fit statistics for the implemented models were performed calculating marginal and condition pseudo‐*R* ^2^ as implemented in R package *piecewiseSEM* (version 1.1.3; Lefcheck, 2015) based on Nakagawa and Schielzeth (2013). All statistical analyses were performed in R version 3.2.0 (R Development Core Team, 2015).

## 3 Results

### 3.1 Diversification along an elevation gradient

**Figure 2:**
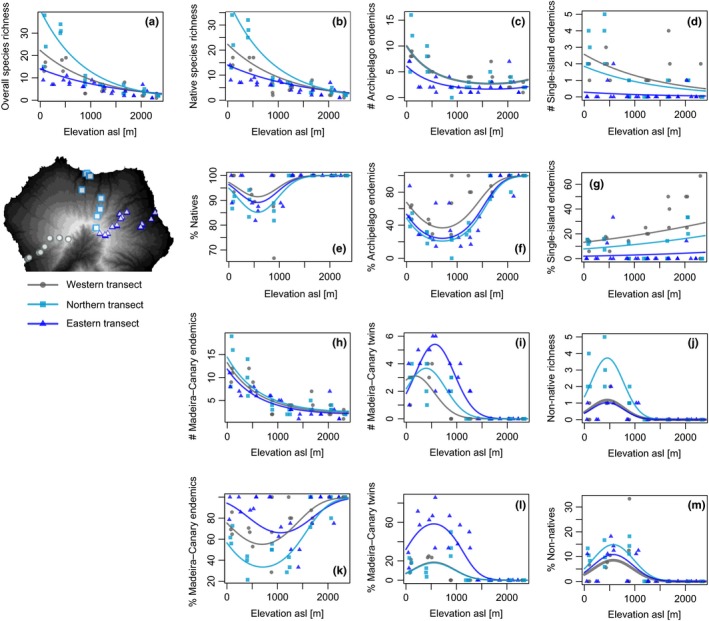
Total species richness (a), native species richness (b), single‐island and archipelago endemic richness (c and d), their respective percentage values (e–g). The number of species endemic to Madeira and the Canary archipelago (including Canary only endemics, h), as well as Madeira–Canary twins, which are endemic to both island groups (excluding Canary only endemics) (i) and non‐natives species richness (j) as well as their percentages (k–m). Regression lines result from generalized linear mixed effect models

Plant species richness showed a highly significant decrease with increase in elevation (*p* < .001). The northern transect showed both higher species richness (*p *>* *.01) and a steeper slope (Figure 2a), as indicated by a significant interaction with elevation (*p *>* *.05). Plant species richness decreased from values at the lowest elevation (approx. 100 m a.s.l.). Richness values reached from more than 30 species on the northern through around half of that on the western and eastern transects to less than three species in the summit region (approx. 2,300 m elevation). Trends remained similar if native species richness was analyzed (non‐native species not included), indicating their high share in total species numbers (Figure 2b).

Canary archipelago endemic richness was low at mid‐ and high elevations with fewer than four species per plot. Canary archipelago endemic richness generally decreased with elevation for all transects, with similar declines from about ten species at 100 m elevation to about four species at the summit (*p *<* *.001 for elevation, *p *<* *.01 for elevation^2^, no interaction between elevation and transect, Figure 2c). The number of Canary archipelago endemics was significantly higher on the northern and western transects, with the difference being about two to three species. This difference disappears if the definition of endemism includes shared Canary–Madeira archipelago endemics (Figure 2h).

The percentage of Canary archipelago endemics displayed a minimum around 750 m for all transects (*p *<* *.001) with overall highest percentage values (100%) in high elevations. The western transect expressed on average higher percentage values than the northern and eastern ones (Figure 2f, *p *<* *.001). The pattern switches if the definition of endemism included Madeira. The proportion of Canary–Madeira archipelago endemics is highest in the eastern transect followed by the western transect (*p *<* *.01). The percentage of Madeira–Canary archipelago endemics shows a mid‐elevation minimum at 1,000–1,400 m interacting with an overall increase with elevation for the western and northern transects that is not present on the eastern transect (interaction *p *<* *.05, Figure 2k). The eastern transect also differed by showing a much higher proportion of endemics reaching up to 80%–100%.

The overall number of single‐island endemic species is low (*n *=* *14), making their interpretation tentative. Single‐island endemic richness per plot showed significant differences between transects (no interaction), slightly declined on the western transect and increased on the northern transect (*p *<* *.01) but did not change along the elevation gradient on the eastern transect. The number of single‐island endemics per plot increased from the eastern transect through the northern transect to the western transect (*p *<* *.001, Figure 2d). Overall, the western transect had nine and the northern transect eight single‐island endemic species compared to four species on the eastern transect.

The percentage of single‐island endemics differed significantly between transects, with the western transect having the highest values. The percentage of single‐island endemic species showed an increase with elevation for the western and northern transects but not for the eastern transect (overall *p *<* *.05, elevation^2^, Figure 2g).

Canary–Madeira twins (endemic species occurring on Madeira and the Canary Islands) showed a unimodal distribution that peaked at different locations along the elevational gradient (*p *<* *.01 for elevation, *p *<* *.001 for elevation^2^, interaction *p *<* *.05, Figure 2i). For the western transect, the relationship peaked at low elevations, while on the northern and eastern transects, the relationship peaked shortly below or above 500 m elevation, respectively.

The share of Canary–Madeira twins in the Canary flora was highest at mid‐elevations (*p *<* *.001, no interaction, Figure 2l). As expected, the percentage was highest on the eastern transect, which transcends the cloud forest zone (*p *<* *.001).

### 3.2 Invasion along elevational gradients

Non‐native species richness reached its maximum around 500 m (Figure 2j) and afterward decreased for all transects with elevation (*p *<* *.001, no interactions), with an above average number of non‐native species on the northern slopes. Non‐native species richness ranged from zero to five species on the northern transect and zero to two in the western and eastern transects. Above an elevation of approximately 1,200 m, non‐native species were missing. The percentage of non‐natives displayed a similar maximum at 500 m followed by a decrease with elevation (*p *<* *.05), without differences between island aspect (Figure 2m). The percentage of non‐natives spanned from 10% at 500 m to zero around 1,200 m and above.

## 4 Discussion

### 4.1 Species diversity and diversification along elevational gradients

In our study, plant species richness as well as the number of archipelago (Canary and Canary–Madeira) endemic species decreased with elevation, a common pattern which was previously found on La Palma (Irl, Steinbauer, Epperlein, et al., 2014) and other islands (Trigas, Panitsa, & Tsiftsis, 2013). A decrease in surface area of individual elevational zones with increase in altitude is made responsible for this effect (Lomolino, 2000; but see Elsen & Tingley, 2015). Furthermore, area is often seen as a surrogate for habitat diversity (Hortal et al., 2009). Larger areas might support larger population sizes, which in turn decreases the risk of extinction (MacArthur, 1972). However, as we used a standardized sampling design with equally sized plots, there is no effect of (plot) area on species richness in our study. Rather, local species richness may be enhanced due to “sampling” from a larger species pool generally available at low elevations (Karger et al., 2011). Harsh environmental conditions with climatic extreme events at high elevations might additionally decrease total and native species richness and density (habitat harshness hypothesis sensu Defeo, Gomez, & Lercari, 2001).

The geographic isolation that reduces the number of individuals pre‐adapted to high‐elevation ecosystems arriving on an island may enhance diversification by genetic isolation (nonadaptive allopatric speciation). Species communities filtered by isolation may further provide ecological opportunity for adaptive radiation to fill gaps in resource usage that are left unoccupied (Steinbauer et al., 2016). Opportunity‐driven diversification is supported by phylogenetic studies (Bennett & O'Grady, 2013). Both mechanisms can help to explain the increase in the percentage of Canary archipelago endemics as well as the percentage of single‐island endemics with elevation, expressing a higher degree of endemism in high‐elevation ecosystems (Steinbauer, Otto, Naranjo‐Cigala, Beierkuhnlein, & Fernández‐Palacios, 2012; Verboom, Bergh, Haiden, Hoffmann, & Britton, 2015). In contrast to richness‐based indices (like the number of endemics), the percentage of endemic species indicates to which degree local species pools on oceanic islands originate from in situ speciation (the alternative origin being colonization from other areas, Irl et al., 2015).

In summary, two explanations for the observed pattern in the three elevational gradients are in accordance with the suggested relative immaturity of high‐elevation ecosystems compared to lower elevations (Fernández‐Palacios et al., 2014). First, in lower elevations, species cannot realize their complete potential distribution due to biotic interactions. Such competitive interactions, however, decrease with elevation, which is particularly true for high‐elevation ecosystems owing to their low species richness (but see Irl et al., 2012). Second, the spatial isolation of high‐elevation species or clades provides the possibility for diversification (speciation enhanced by isolation as well as by ecological opportunity; Steinbauer et al., 2016). The immigration of populations from other islands decreases with elevation due to longer distances to source regions and smaller areas (Merckx et al., 2015; Steinbauer et al., 2013). In addition, locally disturbances are an additional key factor influencing the higher percentage of Canary archipelago and single‐island endemics. Particularly, plots in the lower parts of the western and northern transects (0–800 m a.s.l.) have a high fraction of native species that are associated with habitats modified and/or disturbed by humans (ruderal species), which might influence the percentage of endemics.

On the eastern transect, which is better preserved compared to the northern transect (especially the laurel forest), the proportion of Canary–Madeira endemics does not increase with elevation. In contrast, single‐island endemic species are virtually absent from this transect, not showing differences between lower and higher elevations, and Canary archipelago endemics richness is low. One reason for this may be the fact that the laurel forest shares a large number of floral elements with Madeira (as is reflected in the number of Canary–Madeira archipelago endemics; Figure 2i). This highlights that dispersal vectors (and thus isolation) differ between the more humid laurophyllous eastern vegetation and the drier western parts of La Palma indicating that dispersal characteristics vary between different vegetation units even within a single island (Nogales et al., 2015). However, future studies will need to identify whether dispersal traits differ between vegetation zones on islands and how this influences diversification.

Finally, ecological isolation, not only geographical isolation, might be responsible for the high diversification in the laurel forests. Since the onset of the Mediterranean climate, laurel forests only persisted in Macaronesia, where the oceanic environment buffered the climatic oscillations of the Pleistocene (Fernández‐Palacios et al., 2011; Patiño et al., 2016). In summary, we suggest that environmental filtering, anthropogenic disturbance, and spatial and ecological isolation of communities to be the main factors responsible for the observed changes in spatial trends with elevation.

### 4.2 Elevational threshold for non‐natives along the elevational gradients

Non‐native species richness on La Palma shows a clear peak at 500 m, probably associated with the main centers of population and agriculture in this elevational zone (del Arco Aguilar, González‐González, Garzón‐Machado, & Pizarro‐Hernández, 2010). Beyond that, elevation exhibits a strong negative influence on non‐native species richness and the percentage of non‐native species. Above about 1,200 m a.s.l., non‐native species were missing in this study. Environmental conditions above this threshold seem to be unfavorable for non‐native plant species on La Palma, at least at this state of the (dynamic) invasion process. In the following, we make several assumptions for the existence of this threshold:

1. The 1,200‐m threshold corresponds very well with the transition from cultivated lowland vegetation (i.e., plots located mainly in extensively managed rangeland or recently abandoned cultivation) to the pine forests (although cultivation can reach higher elevations in other areas of La Palma). Above 1,200 m elevation, only extensive forestry is practiced in the sampled area due to increasing steepness and deteriorating climatic conditions. As a consequence, human disturbances associated with agriculture and the probability of an anthropogenic introduction of non‐native species are greatly reduced above the 1,200‐m threshold. However, historic land use in form of herding, fire, and hunting has affected and still affects the whole environmental gradient (Garzón‐Machado et al., 2010; Irl, Steinbauer, Messinger, et al., 2014).
2. *Pinus canariensis* forests are highly dominant above this threshold (1,200–1,500 m). This vegetation type has the highest fire frequency on the Canary Islands. Only non‐native species with a relatively high fire tolerance can potentially establish in the vegetation zone of the pine forest; thus, fire might act as a strong filter for potential invaders particularly as many fire‐prone ecosystems in other parts of the world are nonforested. However, fire can also cause a short‐term increase in native and non‐native annual plants (mostly ruderals; González Gómez et al., 2011). At high elevations, fire together with harsh conditions (Kueffer, Pyšek, & Richardson, 2013), such as high solar radiation, strong diurnal temperature amplitude, extensive summer aridity, and winter snow and ice storms (Garzón‐Machado et al., 2014), probably filter potential invaders in the summit scrub.
3. The sampling design of this study avoided plant communities of pure human origin such as roadside communities, which are often applied for questions regarding plant invasion in mountain systems (see, e.g., Alexander et al., 2011; Arévalo et al., 2010; Otto et al., 2014). Roads have been shown to be very effective vectors of plant invasion and homogenization on the neighboring islands of Tenerife (Arévalo et al., 2010; Haider et al., 2010) and in other mountainous areas of the world (Alexander et al., 2011). Roadside communities at higher elevations exist on La Palma as well, where non‐native species such as *Reseda luteola* L. reach up into the highest elevations (unpublished data). In general, roadside communities might help to identify maximum elevations reached by non‐native species, indicating physiological limits of non‐natives (e.g., temperature thresholds; Poll, Naylor, Alexander, Edwards, & Dietz, 2009), yet focusing on more natural communities is a better tool to determine the state of invasibility and might help to identify how biological constraints such as interspecific competition limit plant invasion.

Introduced plants tend to be pre‐adapted to low‐elevation sites because introduction usually takes place at low elevations. The low amount of non‐native high‐elevation specialists and the filtering effect of the *P. canariensis* forest (Alexander et al., 2011) lead to the absence of non‐native species in high‐elevation ecosystems. However, this may be only a transitional situation, as global climate change has the potential to alter the high‐elevation harshness filter on islands, creating possibilities for invasion at increasing elevations in the near future (Harter et al., 2015).

Besides increasing temperatures, increasing moisture availability has been shown to facilitate the invasion process (Maron & Marler, 2007; Stohlgren et al., 1999). We observed highest values for non‐native species richness on the northern transect. This supports environmental filtering resulting in lower non‐native species richness on the western side of the island. On the other hand, the dense evergreen canopy of the laurel forest on the eastern transect prevents most light from penetrating to the ground (Delgado, Arroyo, Arévalo, & Fernández‐Palacios, 2007), which potentially might prevent the establishment of heliophyllous non‐native species. Generally, the total number of non‐native plants and their contribution to the vegetation structure and composition are low on La Palma compared with many oceanic islands (Daehler, 2005; Macdonald, Thébaud, Strahm, & Strasberg, 1991). This fact, together with the identified spatial patterns, may contribute to coping and management strategies in order to mitigate possible future negative impacts of invasive species at an early stage.

### 4.3 Theoretical concept of invasion and speciation on oceanic islands

Invasion of non‐native plant species and diversification of native and endemic plants are generally seen as two independent processes. However, the underlying environmental drivers on oceanic islands' elevational gradients, that is, environmental filtering, degree of human influence, and geographic filtering in the form of spatial and ecological isolation, seem to influence both processes, but in contrasting directions. Aiming to dissect the presumed interrelation of underlying environmental drivers with these processes, Levin (2003) suggested using islands as research objects because of their excellent setting for this purpose.

Consistent with phylogenetic findings (Merckx et al., 2015), Steinbauer et al. (2016) found elevation‐driven isolation to positively influence speciation. This seems to be a general phenomenon on high‐elevation islands but also applies to mountains on the continent, highlighting its importance for global biodiversity patterns. Although anthropogenic influences affect current species distribution patterns, for example, via the occurrence of ruderal species, the evolutionary signal is still strong in the spatial gradients detected in our study. The lower immigration rates at higher elevations results in small species pools, whereas the degree of endemism, as a proxy for diversification, is high (Merckx et al., 2015). A combination of drivers effectively impedes colonization of high elevations by natural immigration and colonization. Among these drivers are harsh climatic conditions, decreasing area and isolation caused by the effective geographical isolation from comparable ecosystems (Steinbauer et al., 2013, 2016).

At the current stage of invasion, high‐elevation ecosystems seem to be better protected from invasion processes than low elevations on La Palma. Recent syntheses show that this is a general phenomenon because harshness tends to decrease the invasibility of a system (*invasion syndrome*; Kueffer et al., 2013). However, changes in land use and climate in the near future might alter invasion filters at high elevations, posing a threat to these environments (Alexander et al., 2016). While many lowland systems are presumably nonrestorable, high‐elevation systems still offer the opportunity to prevent large‐scale alterations by non‐native species, if immediate protection measures are taken.

## Conflict of Interest

None declared.
