# Supplementary material for: Plant invasion and speciation along elevational gradients on the oceanic island La Palma, Canary Islands
Source: Ecol Evol. 2016 Dec 27;7(2):771–9. doi: 10.1002/ece3.2640 (PMC5243188; doi:10.1002/ece3.2640)
Supplement: Supplementary file 1 [file ECE3-7-771-s001.docx]

Supporting information for manuscript Steinbauer MJ, Irl SDH, González-Mancebo JM, Breiner FT, Hernández RH, Hopfenmüller S, Kidane Y, Jentsch A, Beierkuhnlein C. Plant invasion and speciation along elevational gradients on the oceanic island La Palma, Canary Islands. Ecology and Evolution.

| **Table 1:** Plot elevation and coordinates | | | | |
| --- | --- | --- | --- | --- |
| **Plot** | **Transect** | **Elevation [m]** | **UTM X** | **UTM Y** |
| 1 | w | 78 | 209500 | 3178700 |
| 2 | w | 102 | 209600 | 3178700 |
| 3 | w | 99 | 209600 | 3178800 |
| 4 | w | 430 | 210300 | 3179100 |
| 5 | w | 452 | 210400 | 3179200 |
| 6 | w | 510 | 210600 | 3179300 |
| 7 | w | 893 | 212300 | 3180200 |
| 8 | w | 888 | 212100 | 3180100 |
| 9 | w | 882 | 212400 | 3180200 |
| 10 | w | 1222 | 213000 | 3180900 |
| 11 | w | 1248 | 213000 | 3181000 |
| 12 | w | 1275 | 213100 | 3181100 |
| 13 | w | 1694 | 214100 | 3182300 |
| 14 | w | 1670 | 214000 | 3182400 |
| 15 | w | 1685 | 213900 | 3182500 |
| 16 | w | 2047 | 215500 | 3182400 |
| 17 | w | 2040 | 215500 | 3182500 |
| 18 | w | 2022 | 215400 | 3182500 |
| 19 | w | 2306 | 216900 | 3182400 |
| 20 | w | 2302 | 216900 | 3182400 |
| 21 | w | 2307 | 216900 | 3182300 |
| 22 | n | 86 | 220000 | 3193000 |
| 23 | n | 67 | 219700 | 3192900 |
| 24 | n | 109 | 219800 | 3192900 |
| 25 | n | 402 | 220200 | 3192500 |
| 26 | n | 398 | 220300 | 3192600 |
| 27 | n | 409 | 220400 | 3192500 |
| 28 | n | 893 | 219000 | 3190300 |
| 29 | n | 887 | 219100 | 3190300 |
| 30 | n | 891 | 219100 | 3190200 |
| 31 | n | 1186 | 222100 | 3189000 |
| 32 | n | 1227 | 222300 | 3188900 |
| 33 | n | 1242 | 222200 | 3188900 |
| 34 | n | 1665 | 222000 | 3187300 |
| 35 | n | 1647 | 222000 | 3187300 |
| 36 | n | 1652 | 222000 | 3187100 |
| 37 | n | 2054 | 221600 | 3186100 |
| 38 | n | 2059 | 221600 | 3186000 |
| 39 | n | 2063 | 221500 | 3186000 |
| 40 | n | 2340 | 221300 | 3184100 |
| 41 | n | 2336 | 221400 | 3184200 |
| 42 | n | 2342 | 221400 | 3184100 |
| 43 | e | 47 | 230900 | 3186700 |
| 44 | e | 60 | 229400 | 3187700 |
| 45 | e | 236 | 230300 | 3185600 |
| 46 | e | 270 | 230300 | 3185600 |
| 47 | e | 526 | 229000 | 3186600 |
| 48 | e | 460 | 228800 | 3184900 |
| 49 | e | 581 | 228500 | 3184300 |
| 50 | e | 577 | 228600 | 3184400 |
| 51 | e | 864 | 225200 | 3186500 |
| 52 | e | 842 | 225200 | 3186500 |
| 53 | e | 1026 | 225700 | 3187700 |
| 54 | e | 1070 | 226300 | 3186800 |
| 55 | e | 1255 | 225500 | 3186000 |
| 56 | e | 1270 | 225600 | 3186000 |
| 57 | e | 1414 | 225300 | 3185800 |
| 58 | e | 1438 | 225300 | 3185700 |
| 59 | e | 1685 | 224700 | 3184600 |
| 60 | e | 1662 | 224700 | 3184700 |
| 61 | e | 1855 | 224000 | 3184400 |
| 62 | e | 1853 | 224000 | 3184400 |
| 63 | e | 2118 | 223200 | 3183300 |
| 64 | e | 2030 | 223300 | 3183300 |
| 65 | e | 2217 | 222900 | 3183000 |
| 66 | e | 2217 | 222800 | 3183000 |

| **Table 2:** Species list and classification: **NS & NP**: native sure and native probably; **EA**: Canary Archipelago endemic; **EP**: La Palma endemic; **CM**: Canary-Madeira Twin; **IS & II** : Introduced sure and introduced invasive | |
| --- | --- |
| **Species name** | **Group** |
| *Achyranthes aspera* | IS |
| *Adenocarpus foliolosus* | EA |
| *Adenocarpus viscosus* | EA |
| *Aeonium arboreum* | EA |
| *Aeonium canariense* | EA |
| *Aeonium davidbramwellii* | EP |
| *Aeonium goochiae* | EP |
| *Aeonium nobile* | EP |
| *Ageratina adenophora* | II |
| *Ageratina riparia* | II |
| *Aichryson pachycaulon* | EA |
| *Aira sp.* | NP |
| *Allium subhirsutum* | NP |
| *Anagallis arvensis* | NP |
| *Anisantha madritensis* | NP |
| *Apollonias barbujana* | CM |
| *Arenaria serpyllifolia* | NP |
| *Argyranthemum frutescens* | EA |
| *Argyranthemum webbii* | EP |
| *Arrhenatherum calderae* | EA |
| *Artemisia thuscula* | EA |
| *Asparagus scoparius* | NP |
| *Asparagus umbellatus* | NS |
| *Asphodelus ramosus* | NP |
| *Bidens pilosa* | IS |
| *Bituminaria bituminosa* | NP |
| *Briza maxima* | NP |
| *Briza minor* | NP |
| *Bystropogon origanifolius* | EA |
| *Calamintha sylvatica* | NP |
| *Calendula arvensis* | NP |
| *Canarina canariensis* | EA |
| *Cardamine hirsuta* | NP |
| *Carduus clavulatus* | EA |
| *Carlina falcata* | EA |
| *Castanea sativa* | IS |
| *Cedronella canariensis* | CM |
| *Cenchrus ciliaris* | NP |
| *Ceropegia dichotoma* | EA |
| *Chamaecytisus proliferus* | EA |
| *Cistus monspeliensis* | NS |
| *Cistus symphytifolius* | EA |
| *Convolvulus althaeoides* | NP |
| *Crambe santosii* | EP |
| *Cuscuta planiflora* | NP |
| *Cynosurus echinatus* | NP |
| *Cytinus hypocistis* | NP |
| *Daucus carota* | NP |
| *Descurainia gilva* | EP |
| *Descurainia millefolia* | EA |
| *Echium bethencourtii* | EP |
| *Echium brevirame* | EP |
| *Echium gentianoides* | EP |
| *Erica arborea* | NS |
| *Erodium malacoides* | NP |
| *Erysimum scoparium* | EA |
| *Euphorbia canariensis* | EA |
| *Euphorbia lamarckii* | EA |
| *Euphorbia peplis* | NP |
| *Foeniculum vulgare* | NP |
| *Fumaria muralis* | NP |
| *Galium scabrum* | NP |
| *Galium verrucosum* | NP |
| *Geranium purpureum* | NP |
| *Geranium rotundifolium* | NP |
| *Globularia salicina* | CM |
| *Gonospermum canariense* | EA |
| *Hedera canariensis* | EA |
| *Holcus lanatus* | NP |
| *Hyparrhenia hirta* | NS |
| *Hypericum canariensis* | CM |
| *Hypericum glandulosum* | CM |
| *Hypericum grandifolium* | CM |
| *Ilex canariensis* | CM |
| *Ixanthus viscosus* | EA |
| *Jasminum odoratissimum* | CM |
| *Kleinia neriifolia* | EA |
| *Lactuca palmensis* | EP |
| *Lathyrus articulatus* | IP |
| *Lathyrus sphaericus* | NP |
| *Laurus novocanariensis* | CM |
| *Lavandula canariensis* | EA |
| *Linum strictum* | NP |
| *Lobularia canariensis* | NS |
| *Lotus hillebrandii* | EP |
| *Malva parviflora* | NP |
| *Maytenus canariensis* | EA |
| *Medicago polymorpha* | NP |
| *Mercurialis annua* | IP |
| *Micromeria herpyllomorpha* | EP |
| *Morella faya* | NS |
| *Ocotea foetens* | CM |
| *Opuntia dillenii* | II |
| *Opuntia maxima* | II |
| *Opuntia tomentosa* | II |
| *Parietaria debilis* | NP |
| *Paronychia canariensis* | EA |
| *Pericallis apendiculata* | EA |
| *Pericallis papyracea* | EP |
| *Periploca laevigata* | CM |
| *Persea indica* | CM |
| *Phagnalon saxatile* | NP |
| *Phyllis nobla* | CM |
| *Picconia excelsa* | CM |
| *Pinus canariensis* | EA |
| *Plantago lagopus* | NP |
| *Plantago webbii* | EA |
| *Polycarpaea smithii* | EA |
| *Prunus lusitanica* | NS |
| *Reichardia ligulata* | EA |
| *Rhamnus crenulata* | EA |
| *Rubia fruticosa* | CM |
| *Rubia peregrina* | NS |
| *Rubus bollei* | EA |
| *Rubus ulmifolius* | NP |
| *Rumex bucephalophorus* | NS |
| *Rumex lunaria* | EA |
| *Salvia canariensis* | EA |
| *Schizogyne sericea* | CM |
| *Scrophularia glabrata* | EA |
| *Semele androgyna* | CM |
| *Sherardia arvensis* | NP |
| *Silene gallica* | NP |
| *Smilax canariensis* | CM |
| *Sonchus hierrensis* | EA |
| *Sonchus palmensis* | EP |
| *Spartocytisus supranubius* | EA |
| *Tamus edulis* | NS |
| *Teline stenopetala* | NS |
| *Todaroa montana* | EA |
| *Tolpis barbata* | NP |
| *Trifolium angustifolium* | NP |
| *Trifolium arvense* | NP |
| *Trifolium campestre* | NP |
| *Umbilicus gaditanus* | NP |
| *Viburnum rigidum* | EA |
| *Vicia tetrasperma* | NP |
| *Visnea mocanera* | CM |
| *Vulpia bromoides* | NP |

| **Table 3:** Species occurrence per plot | |
| --- | --- |
| **plot** | ***species*** |
| 1 | *Aeonium canariensis* |
| 1 | *Anisantha madritensis* |
| 1 | *Bituminaria bituminosa* |
| 1 | *Cenchrus ciliaris* |
| 1 | *Echium brevirame* |
| 1 | *Euphorbia lamarckii* |
| 1 | *Kleinia neriifolia* |
| 1 | *Lavandula canariensis* |
| 1 | *Micromeria herpyllomorpha* |
| 1 | *Periploca laevigata* |
| 1 | *Phagnalon saxatile* |
| 1 | *Reichardia ligulata* |
| 1 | *Rumex lunaria* |
| 2 | *Anisantha madritensis* |
| 2 | *Bituminaria bituminosa* |
| 2 | *Cenchrus ciliaris* |
| 2 | *Echium brevirame* |
| 2 | *Euphorbia canariensis* |
| 2 | *Euphorbia lamarckii* |
| 2 | *Kleinia neriifolia* |
| 2 | *Lavandula canariensis* |
| 2 | *Lobularia canariensis* |
| 2 | *Malva parviflora* |
| 2 | *Periploca laevigata* |
| 2 | *Phagnalon saxatile* |
| 2 | *Reichardia ligulata* |
| 2 | *Rubia fruticosa* |
| 2 | *Rumex lunaria* |
| 2 | *Salvia canariensis* |
| 2 | *Schizogyne sericea* |
| 3 | *Anisantha madritensis* |
| 3 | *Echium brevirame* |
| 3 | *Euphorbia canariensis* |
| 3 | *Euphorbia lamarckii* |
| 3 | *Kleinia neriifolia* |
| 3 | *Lavandula canariensis* |
| 3 | *Micromeria herpyllomorpha* |
| 3 | *Opuntia dillenii* |
| 3 | *Periploca laevigata* |
| 3 | *Phagnalon saxatile* |
| 3 | *Polycarpaea smithii* |
| 3 | *Reichardia ligulata* |
| 3 | *Rubia fruticosa* |
| 3 | *Rumex lunaria* |
| 3 | *Schizogyne sericea* |
| 4 | *Aeonium davidbramwellii* |
| 4 | *Aeonium goochiae* |
| 4 | *Aeonium canariensis* |
| 4 | *Anisantha madritensis* |
| 4 | *Bituminaria bituminosa* |
| 4 | *Cenchrus ciliaris* |
| 4 | *Cheilanthes maderensis* |
| 4 | *Euphorbia lamarckii* |
| 4 | *Globularia salicina* |
| 4 | *Kleinia neriifolia* |
| 4 | *Lavandula canariensis* |
| 4 | *Medicago polymorpha* |
| 4 | *Micromeria herpyllomorpha* |
| 4 | *Opuntia dillenii* |
| 4 | *Periploca laevigata* |
| 4 | *Phagnalon saxatile* |
| 4 | *Pinus canariensis* |
| 4 | *Rubia fruticosa* |
| 4 | *Schizogyne sericea* |
| 5 | *Aeonium nobile* |
| 5 | *Anisantha madritensis* |
| 5 | *Bituminaria bituminosa* |
| 5 | *Cenchrus ciliaris* |
| 5 | *Euphorbia lamarckii* |
| 5 | *Globularia salicina* |
| 5 | *Kleinia neriifolia* |
| 5 | *Lavandula canariensis* |
| 5 | *Medicago polymorpha* |
| 5 | *Micromeria herpyllomorpha* |
| 5 | *Opuntia dillenii* |
| 5 | *Periploca laevigata* |
| 5 | *Rubia fruticosa* |
| 6 | *Anisantha madritensis* |
| 6 | *Asparagus scoparius* |
| 6 | *Bituminaria bituminosa* |
| 6 | *Calendula arvensis* |
| 6 | *Carlina falcata* |
| 6 | *Convolvulus althaeoides* |
| 6 | *Euphorbia lamarckii* |
| 6 | *Foeniculum vulgare* |
| 6 | *Globularia salicina* |
| 6 | *Micromeria herpyllomorpha* |
| 6 | *Opuntia dillenii* |
| 6 | *Opuntia maxima* |
| 6 | *Periploca laevigata* |
| 6 | *Phagnalon saxatile* |
| 6 | *Pinus canariensis* |
| 6 | *Plantago lagopus* |
| 6 | *Rubia fruticosa* |
| 6 | *Sonchus hierrensis* |
| 6 | *Vicia tetrasperma* |
| 7 | *Cistus symphytifolius* |
| 7 | *Lathyrus articulatus* |
| 7 | *Pinus canariensis* |
| 8 | *Cistus monspeliensis* |
| 8 | *Cistus symphytifolius* |
| 8 | *Pinus canariensis* |
| 9 | *Allium subhirsutum* |
| 9 | *Bituminaria bituminosa* |
| 9 | *Cistus monspeliensis* |
| 9 | *Geranium purpureum* |
| 9 | *Lathyrus articulatus* |
| 9 | *Pericallis papyracea* |
| 9 | *Pinus canariensis* |
| 9 | *Vicia tetrasperma* |
| 10 | *Adenocarpus foliolosus* |
| 10 | *Cistus symphytifolius* |
| 10 | *Lotus hillebrandii* |
| 10 | *Pinus canariensis* |
| 11 | *Adenocarpus foliolosus* |
| 11 | *Cistus symphytifolius* |
| 11 | *Lotus hillebrandii* |
| 11 | *Pinus canariensis* |
| 11 | *Vicia tetrasperma* |
| 12 | *Adenocarpus foliolosus* |
| 12 | *Cistus symphytifolius* |
| 12 | *Lotus hillebrandii* |
| 12 | *Pinus canariensis* |
| 12 | *Vicia tetrasperma* |
| 13 | *Adenocarpus foliolosus* |
| 13 | *Descurainia gilva* |
| 13 | *Lactuca palmensis* |
| 13 | *Pinus canariensis* |
| 13 | *Vicia tetrasperma* |
| 14 | *Adenocarpus foliolosus* |
| 14 | *Cistus symphytifolius* |
| 14 | *Descurainia gilva* |
| 14 | *Lactuca palmensis* |
| 14 | *Lotus hillebrandii* |
| 14 | *Micromeria herpyllomorpha* |
| 14 | *Pinus canariensis* |
| 14 | *Vicia tetrasperma* |
| 15 | *Adenocarpus foliolosus* |
| 15 | *Descurainia gilva* |
| 15 | *Pinus canariensis* |
| 15 | *Vicia tetrasperma* |
| 16 | *Adenocarpus viscosus* |
| 16 | *Descurainia gilva* |
| 17 | *Adenocarpus viscosus* |
| 17 | *Descurainia gilva* |
| 17 | *Pinus canariensis* |
| 17 | *Scrophularia glabrata* |
| 18 | *Adenocarpus viscosus* |
| 18 | *Descurainia gilva* |
| 19 | *Adenocarpus viscosus* |
| 19 | *Descurainia gilva* |
| 19 | *Lactuca palmensis* |
| 20 | *Adenocarpus viscosus* |
| 21 | *Adenocarpus viscosus* |
| 21 | *Plantago webbii* |
| 22 | *Aeonium arboreum* |
| 22 | *Aeonium goochiae* |
| 22 | *Aeonium canariensis* |
| 22 | *Allium subhirsutum* |
| 22 | *Anagallis arvensis* |
| 22 | *Argyranthemum frutescens* |
| 22 | *Asphodelus ramosus* |
| 22 | *Bidens pilosa* |
| 22 | *Bituminaria bituminosa* |
| 22 | *Briza maxima* |
| 22 | *Calendula arvensis* |
| 22 | *Carduus clavulatus* |
| 22 | *Echium bethencourtii* |
| 22 | *Erodium malacoides* |
| 22 | *Euphorbia canariensis* |
| 22 | *Euphorbia lamarckii* |
| 22 | *Euphorbia peplis* |
| 22 | *Holcus lanatus* |
| 22 | *Hypericum glandulosum* |
| 22 | *Kleinia neriifolia* |
| 22 | *Lavandula canariensis* |
| 22 | *Lobularia canariensis* |
| 22 | *Mercurialis annua* |
| 22 | *Micromeria herpyllomorpha* |
| 22 | *Opuntia maxima* |
| 22 | *Opuntia tomentosa* |
| 22 | *Parietaria debilis* |
| 22 | *Pericallis papyracea* |
| 22 | *Periploca laevigata* |
| 22 | *Reichardia ligulata* |
| 22 | *Rubia fruticosa* |
| 22 | *Rumex lunaria* |
| 22 | *Sonchus hierrensis* |
| 22 | *Todaroa montana* |
| 22 | *Tolpis barbata* |
| 22 | *Trifolium campestre* |
| 22 | *Umbilicus gaditanus* |
| 22 | *Vicia tetrasperma* |
| 23 | *Aeonium goochiae* |
| 23 | *Ageratina adenophora* |
| 23 | *Ageratina riparia* |
| 23 | *Anisantha madritensis* |
| 23 | *Bituminaria bituminosa* |
| 23 | *Euphorbia lamarckii* |
| 23 | *Foeniculum vulgare* |
| 23 | *Fumaria muralis* |
| 23 | *Globularia salicina* |
| 23 | *Hyparrhenia hirta* |
| 23 | *Lavandula canariensis* |
| 23 | *Micromeria herpyllomorpha* |
| 23 | *Periploca laevigata* |
| 23 | *Rubia fruticosa* |
| 23 | *Todaroa montana* |
| 24 | *Aeonium arboreum* |
| 24 | *Aeonium goochiae* |
| 24 | *Aeonium canariensis* |
| 24 | *Anisantha madritensis* |
| 24 | *Bidens pilosa* |
| 24 | *Bituminaria bituminosa* |
| 24 | *Ceropegia dichotoma* |
| 24 | *Cuscuta planiflora* |
| 24 | *Echium bethencourtii* |
| 24 | *Euphorbia canariensis* |
| 24 | *Euphorbia lamarckii* |
| 24 | *Globularia salicina* |
| 24 | *Hyparrhenia hirta* |
| 24 | *Hypericum canariensis* |
| 24 | *Kleinia neriifolia* |
| 24 | *Lavandula canariensis* |
| 24 | *Opuntia maxima* |
| 24 | *Paronychia canariensis* |
| 24 | *Pericallis papyracea* |
| 24 | *Periploca laevigata* |
| 24 | *Phagnalon saxatile* |
| 24 | *Rubia fruticosa* |
| 24 | *Sonchus hierrensis* |
| 24 | *Trifolium arvense* |
| 25 | *Aeonium arboreum* |
| 25 | *Aeonium davidbramwellii* |
| 25 | *Aeonium goochiae* |
| 25 | *Ageratina adenophora* |
| 25 | *Allium subhirsutum* |
| 25 | *Anagallis arvensis* |
| 25 | *Anisantha madritensis* |
| 25 | *Argyranthemum webbii* |
| 25 | *Asparagus scoparius* |
| 25 | *Asphodelus ramosus* |
| 25 | *Bituminaria bituminosa* |
| 25 | *Briza maxima* |
| 25 | *Briza minor* |
| 25 | *Cynosurus echinatus* |
| 25 | *Daucus carota* |
| 25 | *Echium bethencourtii* |
| 25 | *Euphorbia lamarckii* |
| 25 | *Geranium purpureum* |
| 25 | *Geranium rotundifolium* |
| 25 | *Hypericum glandulosum* |
| 25 | *Jasminum odoratissimum* |
| 25 | *Kleinia neriifolia* |
| 25 | *Lavandula canariensis* |
| 25 | *Notholaena marantae* |
| 25 | *Opuntia maxima* |
| 25 | *Pericallis papyracea* |
| 25 | *Phagnalon saxatile* |
| 25 | *Plantago lagopus* |
| 25 | *Rubia fruticosa* |
| 25 | *Rumex bucephalophorus* |
| 25 | *Sherardia arvensis* |
| 25 | *Silene gallica* |
| 25 | *Sonchus hierrensis* |
| 25 | *Tolpis barbata* |
| 25 | *Vicia tetrasperma* |
| 26 | *Achyranthes aspera* |
| 26 | *Aeonium arboreum* |
| 26 | *Aeonium goochiae* |
| 26 | *Aeonium canariensis* |
| 26 | *Argyranthemum webbii* |
| 26 | *Asphodelus ramosus* |
| 26 | *Bidens pilosa* |
| 26 | *Bituminaria bituminosa* |
| 26 | *Briza maxima* |
| 26 | *Cynosurus echinatus* |
| 26 | *Daucus carota* |
| 26 | *Descurainia millefolia* |
| 26 | *Echium bethencourtii* |
| 26 | *Galium verrucosum* |
| 26 | *Hyparrhenia hirta* |
| 26 | *Hypericum glandulosum* |
| 26 | *Lathyrus articulatus* |
| 26 | *Medicago polymorpha* |
| 26 | *Opuntia maxima* |
| 26 | *Opuntia tomentosa* |
| 26 | *Pericallis papyracea* |
| 26 | *Phagnalon saxatile* |
| 26 | *Plantago lagopus* |
| 26 | *Rubia fruticosa* |
| 26 | *Rumex bucephalophorus* |
| 26 | *Silene gallica* |
| 26 | *Sonchus hierrensis* |
| 26 | *Tolpis barbata* |
| 26 | *Umbilicus gaditanus* |
| 26 | *Vicia tetrasperma* |
| 27 | *Aeonium arboreum* |
| 27 | *Aeonium canariensis* |
| 27 | *Ageratina adenophora* |
| 27 | *Allium subhirsutum* |
| 27 | *Anagallis arvensis* |
| 27 | *Anisantha madritensis* |
| 27 | *Asphodelus ramosus* |
| 27 | *Bituminaria bituminosa* |
| 27 | *Briza maxima* |
| 27 | *Calamintha sylvatica* |
| 27 | *Cistus monspeliensis* |
| 27 | *Cytinus hypocistis* |
| 27 | *Erica arborea* |
| 27 | *Hyparrhenia hirta* |
| 27 | *Hypericum glandulosum* |
| 27 | *Kleinia neriifolia* |
| 27 | *Lathyrus articulatus* |
| 27 | *Lathyrus sphaericus* |
| 27 | *Linum strictum* |
| 27 | *Opuntia maxima* |
| 27 | *Pericallis papyracea* |
| 27 | *Phagnalon saxatile* |
| 27 | *Plantago lagopus* |
| 27 | *Sherardia arvensis* |
| 27 | *Sonchus palmensis* |
| 27 | *Tolpis barbata* |
| 27 | *Trifolium angustifolium* |
| 27 | *Trifolium campestre* |
| 27 | *Umbilicus gaditanus* |
| 27 | *Vicia tetrasperma* |
| 27 | *Vulpia bromoides* |
| 28 | *Ageratina riparia* |
| 28 | *Allium subhirsutum* |
| 28 | *Canarina canariensis* |
| 28 | *Castanea sativa* |
| 28 | *Cedronella canariensis* |
| 28 | *Galium scabrum* |
| 28 | *Galium verrucosum* |
| 28 | *Gonospermum canariense* |
| 28 | *Hypericum grandifolium* |
| 28 | *Ilex canariensis* |
| 28 | *Rubia peregrina* |
| 29 | *Ageratina adenophora* |
| 29 | *Asplenium adiantum-nigrum* |
| 29 | *Bituminaria bituminosa* |
| 29 | *Cistus symphytifolius* |
| 29 | *Erica arborea* |
| 29 | *Ilex canariensis* |
| 29 | *Micromeria herpyllomorpha* |
| 29 | *Myrica faya* |
| 29 | *Rubia peregrina* |
| 29 | *Visnea mocanera* |
| 30 | *Ageratina adenophora* |
| 30 | *Cedronella canariensis* |
| 30 | *Erica arborea* |
| 30 | *Hypericum canariensis* |
| 30 | *Laurus novocanariensis* |
| 30 | *Prunus lusitanica* |
| 30 | *Rubia peregrina* |
| 31 | *Asphodelus ramosus* |
| 31 | *Cistus symphytifolius* |
| 31 | *Erica arborea* |
| 31 | *Myrica faya* |
| 31 | *Pinus canariensis* |
| 31 | *Teline stenopetala* |
| 31 | *Vicia tetrasperma* |
| 32 | *Asphodelus ramosus* |
| 32 | *Carlina falcata* |
| 32 | *Cistus symphytifolius* |
| 32 | *Erica arborea* |
| 32 | *Myrica faya* |
| 32 | *Pinus canariensis* |
| 32 | *Vicia tetrasperma* |
| 33 | *Asphodelus ramosus* |
| 33 | *Cistus symphytifolius* |
| 33 | *Erica arborea* |
| 33 | *Geranium purpureum* |
| 33 | *Myrica faya* |
| 33 | *Pinus canariensis* |
| 34 | *Adenocarpus foliolosus* |
| 34 | *Cistus symphytifolius* |
| 34 | *Pinus canariensis* |
| 35 | *Adenocarpus foliolosus* |
| 35 | *Cistus symphytifolius* |
| 35 | *Pinus canariensis* |
| 36 | *Adenocarpus foliolosus* |
| 36 | *Bystropogon origanifolius* |
| 36 | *Cardamine hirsuta* |
| 36 | *Cistus symphytifolius* |
| 36 | *Lactuca palmensis* |
| 36 | *Pinus canariensis* |
| 36 | *Vicia tetrasperma* |
| 37 | *Adenocarpus viscosus* |
| 37 | *Bystropogon origanifolius* |
| 37 | *Lactuca palmensis* |
| 38 | *Adenocarpus viscosus* |
| 38 | *Erysimum scoparium* |
| 38 | *Lactuca palmensis* |
| 39 | *Adenocarpus viscosus* |
| 39 | *Arenaria serpyllifolia* |
| 39 | *Bystropogon origanifolius* |
| 39 | *Lactuca palmensis* |
| 39 | *Pinus canariensis* |
| 40 | *Adenocarpus viscosus* |
| 40 | *Plantago webbii* |
| 41 | *Adenocarpus viscosus* |
| 41 | *Erysimum scoparium* |
| 42 | *Adenocarpus viscosus* |
| 42 | *Erysimum scoparium* |
| 43 | *Aeonium arboreum* |
| 43 | *Artemisia thuscula* |
| 43 | *Asparagus umbellatus* |
| 43 | *Asphodelus ramosus* |
| 43 | *Euphorbia canariensis* |
| 43 | *Euphorbia lamarckii* |
| 43 | *Globularia salicina* |
| 43 | *Hyparrhenia hirta* |
| 43 | *Lavandula canariensis* |
| 43 | *Periploca laevigata* |
| 43 | *Rhamnus crenulata* |
| 43 | *Rubia fruticosa* |
| 43 | *Rumex lunaria* |
| 43 | *Schizogyne sericea* |
| 44 | *Aeonium arboreum* |
| 44 | *Artemisia thuscula* |
| 44 | *Euphorbia canariensis* |
| 44 | *Euphorbia lamarckii* |
| 44 | *Globularia salicina* |
| 44 | *Kleinia neriifolia* |
| 44 | *Lavandula canariensis* |
| 44 | *Opuntia dillenii* |
| 44 | *Rumex lunaria* |
| 45 | *Apollonias barbujana* |
| 45 | *Ilex canariensis* |
| 45 | *Laurus novocanariensis* |
| 45 | *Maytenus canariensis* |
| 45 | *Picconia excelsa* |
| 45 | *Smilax canariensis* |
| 45 | *Viburnum rigidum* |
| 46 | *Apollonias barbujana* |
| 46 | *Asplenium hemionitis* |
| 46 | *Erica arborea* |
| 46 | *Jasminum odoratissimum* |
| 46 | *Laurus novocanariensis* |
| 46 | *Rubus bollei* |
| 46 | *Viburnum rigidum* |
| 46 | *Visnea mocanera* |
| 47 | *Ageratina adenophora* |
| 47 | *Ageratina riparia* |
| 47 | *Hedera canariensis* |
| 47 | *Ilex canariensis* |
| 47 | *Laurus novocanariensis* |
| 47 | *Ocotea foetens* |
| 47 | *Persea indica* |
| 47 | *Phyllis nobla* |
| 47 | *Rubia peregrina* |
| 47 | *Rubus bollei* |
| 47 | *Smilax canariensis* |
| 48 | *Ageratina riparia* |
| 48 | *Hedera canariensis* |
| 48 | *Micromeria herpyllomorpha* |
| 48 | *Ocotea foetens* |
| 48 | *Persea indica* |
| 48 | *Picconia excelsa* |
| 48 | *Tamus edulis* |
| 48 | *Semele androgyna* |
| 48 | *Smilax canariensis* |
| 49 | *Ageratina riparia* |
| 49 | *Apollonias barbujana* |
| 49 | *Hedera canariensis* |
| 49 | *Ilex canariensis* |
| 49 | *Laurus novocanariensis* |
| 49 | *Ocotea foetens* |
| 49 | *Persea indica* |
| 49 | *Picconia excelsa* |
| 50 | *Ageratina riparia* |
| 50 | *Crambe santosii* |
| 50 | *Hedera canariensis* |
| 50 | *Ocotea foetens* |
| 50 | *Pericallis papyracea* |
| 50 | *Persea indica* |
| 50 | *Polystichum setiferum* |
| 50 | *Rubus bollei* |
| 51 | *Hedera canariensis* |
| 51 | *Laurus novocanariensis* |
| 51 | *Pericallis apendiculata* |
| 51 | *Persea indica* |
| 51 | *Picconia excelsa* |
| 51 | *Semele androgyna* |
| 52 | *Aichryson pachycaulon* |
| 52 | *Hedera canariensis* |
| 52 | *Ixanthus viscosus* |
| 52 | *Laurus novocanariensis* |
| 52 | *Pericallis apendiculata* |
| 52 | *Persea indica* |
| 53 | *Ageratina adenophora* |
| 53 | *Erica arborea* |
| 53 | *Hedera canariensis* |
| 53 | *Hypericum grandifolium* |
| 53 | *Ilex canariensis* |
| 53 | *Ixanthus viscosus* |
| 53 | *Laurus novocanariensis* |
| 53 | *Morella faya* |
| 53 | *Rubia peregrina* |
| 54 | *Ageratina riparia* |
| 54 | *Apollonias barbujana* |
| 54 | *Erica arborea* |
| 54 | *Hedera canariensis* |
| 54 | *Ilex canariensis* |
| 54 | *Laurus novocanariensis* |
| 54 | *Morella faya* |
| 54 | *Persea indica* |
| 55 | *Hedera canariensis* |
| 55 | *Laurus novocanariensis* |
| 55 | *Morella faya* |
| 55 | *Persea indica* |
| 56 | *Erica arborea* |
| 56 | *Hedera canariensis* |
| 56 | *Ilex canariensis* |
| 56 | *Laurus novocanariensis* |
| 56 | *Morella faya* |
| 56 | *Rubia peregrina* |
| 57 | *Erica arborea* |
| 57 | *Morella faya* |
| 57 | *Pinus canariensis* |
| 58 | *Adenocarpus foliolosus* |
| 58 | *Erica arborea* |
| 58 | *Morella faya* |
| 58 | *Pinus canariensis* |
| 59 | *Adenocarpus foliolosus* |
| 59 | *Cardamine hirsuta* |
| 59 | *Cistus symphytifolius* |
| 59 | *Pinus canariensis* |
| 60 | *Adenocarpus foliolosus* |
| 60 | *Pinus canariensis* |
| 61 | *Adenocarpus foliolosus* |
| 61 | *Asplenium adiantum-nigrum* |
| 61 | *Pinus canariensis* |
| 62 | *Adenocarpus viscosus* |
| 62 | *Pinus canariensis* |
| 63 | *Adenocarpus viscosus* |
| 63 | *Aira sp.* |
| 63 | *Arrhenatherum calderae* |
| 63 | *Erysimum scoparium* |
| 64 | *Adenocarpus viscosus* |
| 64 | *Arrhenatherum calderae* |
| 64 | *Chamaecytisus proliferus* |
| 64 | *Descurainia millefolia* |
| 64 | *Echium gentianoides* |
| 64 | *Erysimum scoparium* |
| 64 | *Spartocytisus supranubius* |
| 65 | *Adenocarpus viscosus* |
| 66 | *Adenocarpus viscosus* |
